# SIRT1 Mediates the Effects of Sera from Athletes Who Engage in Aerobic Exercise Training in Activating Cells for Wound Healing

**DOI:** 10.3390/biomedicines13051041

**Published:** 2025-04-25

**Authors:** Raffaella Belvedere, Nunzia Novizio, Berenice Stefanelli, Carmine Sellitto, Mariangela Palazzo, Marta Trucillo, Antonio De Luca, Emanuela De Bellis, Graziamaria Corbi, Amelia Filippelli, Valeria Conti, Antonello Petrella

**Affiliations:** 1 Department of Pharmacy, University of Salerno, 84084 Fisciano, Italy; rbelvedere@unisa.it (R.B.); nnovizio@unisa.it (N.N.); marpalazzo@unisa.it (M.P.); apetrella@unisa.it (A.P.); 2 Department of Medicine, Surgery, and Dentistry, University of Salerno, 84081 Baronissi, Italy; bstefanelli@unisa.it (B.S.); afilippelli@unisa.it (A.F.); 3 Clinical Pharmacology Unit, University Hospital “San Giovanni di Dio e Ruggi d’Aragona”, 84131 Salerno, Italy; csellitto@unisa.it; 4 Department of Mental and Physical Health and Preventive Medicine, Section of Human Anatomy, University of Campania “Luigi Vanvitelli”, 80138 Naples, Italy; marta-trucillo@libero.it (M.T.); antonio.deluca@unicampania.it (A.D.L.); 5 PhD School “Clinical and Translational Oncology (CTO)”, Scuola Superiore Meridionale, University of Naples “Federico II”, 80138 Naples, Italy; emanuela.debellis@unina.it; 6 Department of Translational Medical Sciences, University of Naples “Federico II”, 80131 Naples, Italy; graziamaria.corbi@unina.it

**Keywords:** skin wound healing, sirtuins, cell motility, angiogenesis, keratinocytes, fibroblasts, endothelial cells

## Abstract

**Background/Objectives:** Exercise training (ET) can improve wound healing and prevent the recurrence of skin lesions. Aerobic ET stimulates the NAD+-dependent deacetylase sirtuin 1 (SIRT1). The beneficial effects of ET and SIRT1 activation in wound healing have been characterized when considered separately. This study aimed to investigate the potential role of SIRT1 as a mediator of the effects of sera isolated from athletes who regularly participate in aerobic ET (middle-distance running, MDR) on cells primarily involved in wound healing. **Methods:** Human keratinocytes, fibroblasts and endothelial cells were conditioned with sera from middle-distance runners and age-matched sedentary subjects (sed). Cell motility, angiogenesis and the expression of key biomarkers of cell activation were evaluated in the presence or absence of the selective SIRT1 inhibitor EX-527. **Results:** Higher SIRT1 activity was detected in all of the cell lines conditioned with the MDR group sera compared with that in the cells in the sed group sera. The involvement of SIRT1 was demonstrated by EX-527’s selective inhibition. Alongside the increase in SIRT1 activity, a marked increase in migration, invasion and angiogenesis was observed. The levels of E-cadherin decreased while those of integrin β1 and vinculin increased in the keratinocytes and fibroblasts conditioned with the MDR group sera compared to these values with the sed group sera, respectively. Increased levels of differentiation markers, such as involucrin in the keratinocytes, FAP1α in the fibroblasts and CD31 in the endothelial cells, were observed with the MDR group sera compared to these values using the sed group sera. **Conclusions**: The ex vivo/in vitro approach used here links aerobic ET-induced SIRT1 activity to proper tissue regeneration.

## 1. Introduction

Exercise training (ET) is a healthy practice essential in daily life to promote longevity. Although several molecular mediators of exercise-induced repair have been identified, the complete signaling network remains under investigation [1].

The numerous benefits associated with ET include the prevention/mitigation of cardiovascular diseases and metabolic disorders such as obesity, type 2 diabetes and metabolic syndrome and tissue regeneration [2]. In the latter case, it has been reported that ET induces cardiomyocyte proliferation, angiogenesis and mitochondrial biosynthesis and the proliferation of hematopoietic progenitor cells and leukocytes and increases the branching of nerve fibers in the proximal skin and hepatocyte proliferation in patients with a partial hepatectomy.

In addition, exercise induces the regeneration of post-traumatic cartilage lesions and may improve skin wound healing, as demonstrated in animal models and in healthy older adults [3,4].

Among the biological events through which exercise-based interventions could accelerate wound healing times and prevent wound recurrence [5], there is evidence of exercise-induced alterations in nicotinamide adenine dinucleotide (NAD+) homeostasis, which is essential for modulating energy metabolism, molecular signaling processes, epigenetic regulation and DNA repair [6]. In particular, it has been suggested that the increased ATP demand during chronic exercise leads to increased levels of NAD+, which is the cofactor for NAD+-dependent deacetylases called sirtuins [7]. Of the seven members of the sirtuin family, sirtuin 1 (SIRT1) is the best characterized [8]. Several studies have demonstrated its involvement in crucial biological processes mediated by exercise-based interventions with significant effects against inflammation, oxidative stress, energetic homeostasis and senescence [9,10,11].

Sirtuins are involved in skin function and health [12,13]. In particular, in SIRT1 knockout mice, a strong delay in tissue regeneration was found to be associated with a significant decrease in angiogenesis, keratinocyte differentiation and fibroblast recruitment. Additionally, when deprived of SIRT1, human keratinocytes showed a reduction in their migration speed [14]. In contrast, the activation of SIRT1 promoted wound healing by increasing skin cell migration [15], confirming the involvement of SIRT1 in tissue healing. Further evidence reports that this protein is able to restore redox balance and alleviate the course of psoriasis [16] and to inhibit NF-κB-mediated tissue damage in diabetic wound healing [17,18]. SIRT1, activated by resveratrol, has also been shown to regulate angiogenesis, fibroblast proliferation and collagen production [18,19].

In this study, we investigated the potential role of SIRT1 as a mediator of the effects of sera obtained from athletes who engage in regular aerobic exercise training (middle-distance running) on the activation of cells involved in wound healing such as keratinocytes, fibroblasts and endothelial cells.

## 2. Materials and Methods

### 2.1. Ethical Approval

This study received approval from the Campania Sud Ethics Committee (Observational Study no. 86/2020) and was conducted according to the Declaration of Helsinki’s guidelines. All participants signed their informed consent after medical staff explained the aim of and the possible risks associated with this study.

### 2.2. The Study Population

Twenty-three adult competing athletes (nine women and fourteen men) practicing middle-distance running (MDR) and twelve age-matched sedentary volunteers (sed) (six women and six men) participated in the study. The MDR group had practiced middle-distance running for at least 6 months and had received a sports fitness certificate. Neither the MDR group nor the sed group had chronic diseases or had suffered injuries in the last 6 months, and none of them were using drugs or supplements or were following a special diet at the time of enrolment. The main characteristics of the study population are shown in Table 1.

### 2.3. Blood Samples

Blood samples were collected from the antecubital vein under fasting conditions into serum separation vacutainer tubes (SST™) to obtain serum. The serum samples (for the MDR and sed groups) were obtained through centrifugation at 1500× *g* for 10 min. Aliquots of the serum were frozen at −80 °C until the analysis.

### 2.4. Cell Cultures and Treatments

The HaCaT cell line (human immortalized keratinocytes derived from adult male human epidermis; CLS, Cell Lines Service GmbH, Eppelheim, Germany) and the BJ cell line (human immortalized fibroblasts established from skin taken from normal foreskin from a neonatal male; the American Type Culture Collection, ATCC, Manassas, VA, USA) were cultured as previously reported [20]. The HUVEC line (human umbilical vein endothelial cells; the ATCC, Manassas, VA, USA) was derived from a pool of 10 individual donors, as indicated by the company, and was maintained until passage 10. The cells were maintained at 37 °C in a humidified atmosphere at 5% CO_2_–95% air and were serially passed at 70–80% confluence. All of the cells were conditioned for 24 h with the 10% *v*/*v* sera isolated from the sed and MDR groups, with or without the EX-527 SIRT1 inhibitor (Sigma-Aldrich; Saint Louis, MO, USA), dissolved in DMSO and used at a final concentration of 15 µM. As a control (ctrl), we used cells grown only in 10% *v*/*v* FBS.

### 2.5. Western Blotting

The protein expression was examined through SDS-PAGE. Briefly, the total intracellular proteins were extracted from the cells through freezing/thawing them in lysis buffer containing protease inhibitors. The protein content was estimated by adapting the Bradford method using Bio-Rad Dye Reagent Concentrate (BIO-RAD; Hercules, CA, USA) at a ratio of 1:5 in bidistilled water. Bovine serum albumin (BSA; Merck Chemicals; Darmstadt, Germany) was used as reference from a solution of 1 mg/mL in bidistilled water. A total of 20 µg of proteins was loaded onto an 8% electrophoresis gel. Then, the proteins were transferred onto nitrocellulose membranes (Amersham Protran; Little Chalfont, UK), and subsequently, the latter were blocked with 5% p/v non-fat dry milk (BIO-RAD; Hercules, CA, USA) in TBS-Tween 20 (0.1% *v*/*v*; Merck Chemicals; Darmstadt, Germany). The analysis continued with incubation with a rabbit monoclonal primary antibody against SIRT1 (1:1000; #9475 Cell Signaling, Danvers, MS, USA) and a mouse monoclonal anti-tubulin antibody (1:1000; T8328 Sigma-Aldrich, Saint Louis, MO, USA) overnight (O/N) at 4 °C and the related secondary anti-mouse/rabbit HRP-conjugated antibodies. The membranes were exposed to Las4000 (GE Healthcare Life Sciences, Little Chalfont, UK) after incubation for two minutes with homemade ECL solution, and the blots were subsequently obtained.

### 2.6. In Vitro Wound Healing and Invasion Assays

The HaCaT, BJ and HUVEC cells were seeded at 1.5 × 10^5^ per well into a 24-well plastic plate. Once complete cell confluence was reached after 24 h, a wound was made by gently scraping the cells with a sterile plastic p10 pipette tip. To ensure mitosis was blocked, all experimental points were further treated with mitomycin C (10 μg/mL, Sigma-Aldrich; Saint Louis, MO, USA). The wounded cells were photographed through a 4× phase contrast objective lens at time points of 0 and 24 h using a Axiovert 5 microscope with the Axiocam 208 color (Carl Zeiss; Oberkochen, Germany). To determine the migration rate, the distances of every wound border covered were measured.

Cell invasiveness was studied using trans-wells (12 mm diameter, 8.0 pore size; Corning Incorporated, New York, NJ, USA) coated with gelated matrigel (Becton Dickinson Labware; Franklin Lakes, NJ, USA) diluted at 1:3 in serum-free medium, as previously described [21]. The cells were plated in 350 μL of serum-free medium (2 × 10^4^/insert) into the upper chamber of the trans-well. The treatments were performed in 1.4 mL of growth medium in the lower chamber. After 24 h at 37 °C in a 5% CO_2_–95% air humidified atmosphere, the medium was aspirated, and the filters were washed twice with PBS 1× and fixed with p-formaldehyde 4% *v*/*v* in PBS (Lonza, Basel, Switzerland) for 10 min and then with 100% *v*/*v* methanol (Sigma-Aldrich; Saint Louis, MO, USA) for 20 min. At the end, 0.5% *v*/*v* crystal violet prepared from stock crystal violet (powder, Merck Chemicals; Darmstadt, Germany) with distilled water and 20% *v*/*v* methanol (Sigma-Aldrich; Saint Louis, MO, USA) was used to stain the cells on the filters for 15 min. After two washes, the cells on the lower surface were counted in twelve causal fields through the use of an EVOS optical microscope (10×) (Life Technologies Corporation; Carlsbad, CA, USA).

### 2.7. The Tube Formation Assay

A total of 2 × 10^4^ cells/well HUVEC cells were seeded onto a coating of matrigel in the presence or absence of the indicated treatments for each experimental point. After 12 h, images were acquired using the EVOS optical microscope (10×) (Life Technologies Corporation, Carlsbad, CA, USA). Subsequent analyses for both the length of each tube and the number of branches were carried out using ImageJ software 2.14.0 version (NIH, Bethesda, MD, USA; Angiogenesis Analyzer for ImageJ).

### 2.8. Confocal Microscopy

The HaCaT, BJ and HUVEC cells were fixed in p-formaldehyde (4% *v*/*v* in PBS; Lonza, Basel, Switzerland), permeabilized with Triton X-100 0.4% *v*/*v* in PBS (Lonza, Basel, Switzerland) and blocked with goat serum 20% *v*/*v* in PBS (Lonza, Basel, Switzerland). Next, the cells were incubated with rabbit polyclonal anti-CD31 (1:100; Abcam, Cambridge, UK), anti-FAP1α (1:250; Cusabio, Houston, TX, USA), mouse monoclonal anti-E-cadherin (1:100, Santa Cruz Biotechnologies, Dallas, TX, USA), anti-integrin β1 (1:250; Cell Signaling, Danvers, MS, USA), anti-involucrin (1:100; Santa Cruz Biotechnologies, Dallas, TX, USA) and anti-vinculin (1:100; Santa Cruz Biotechnologies, Dallas, TX, USA) in O/N at 4 °C. After two washing steps with PBS, the cells were incubated with anti-rabbit and/or anti-mouse Alexa Fluor (488 and/or 555; 1:500; Molecular Probes, Eugene, OR, USA) for 2 h at RT. The coverslips were mounted in Mowiol (Mowiol 4–88, Sigma-Aldrich, Saint Louis, MO, USA). Nuclei were detected using DAPI (1:1000). The samples were vertically scanned using a TCS SP8 Leica Microsystems microscope (Wetzlar, Germany) with a HC PL APO 63 × (1.40 NA) oil-immersion objective. The mean fluorescence intensity was analyzed using ImageJ software 2.14.0 version (NIH, Bethesda, MD, USA). Values are reported as the normalization between the protein signal and the DAPI signal (in arbitrary units, AU).

### 2.9. SIRT1 Activity

Nuclei were extracted by suspending 1 × 10^7^ cells into 1 mL of lysis buffer (10 mM of Tris HCl at a pH of 7.5, 10 mM of NaCl, 15 mM of MgCl_2_, 250 mM of sucrose, 0.5% *v*/*v* NP-40, and 0.1 mM of EGTA). The cells were spun through 4 mL of a sucrose cushion (30% p/v sucrose, 10 mM of Tris HCl at a pH of 7.5, 10 mM of NaCl, and 3 mM of MgCl_2_) at 1300 g for 10 min at 4 °C. The isolated nuclei were suspended in 50–100 µL of extraction buffer (50 mM of HEPES KOH at a pH of 7.5, 420 mM of NaCl, 0.5 mM of EDTA Na_2_, 0.1 mM of EGTA, and 10% *v*/*v* glycerol). After centrifugation at 15,000× *g* rpm for 10 min, the protein concentration was determined using the Bradford method. SIRT1 activity was determined using a fluorometric deacetylase assay kit specific to SIRT1 (Abcam, Cambridge, UK). The reaction was performed by simultaneously mixing a fluorescent-labeled acetylated peptide as the substrate, 10 µL of the sample, trichostatin A, NAD, and lysyl endopeptidase. The fluorescence intensity at 440 nm was measured 60 min after the start of the reaction in an Enspire 2300 Multimode plate reader (PerkinElmer, Waltham, MA, USA). Values are reported as the relative fluorescence per µg of protein (in arbitrary units, AU).

### 2.10. Statistical Analysis

The data analyses and statistical evaluations were carried out using Microsoft Excel and GraphPad Prism 6 software. The number of independent experiments, the standard deviation (SD), and the p-values are indicated in the figure legends. All of the results are the means ± SDs of at least 3 experiments performed in triplicate. To assess the normality of the distribution of data, the Shapiro–Wilk test was used. To test for homoscedasticity in an ANOVA, Levene’s test was used. Differences between multiple groups were evaluated through an analysis of variance (ANOVA) with the post hoc Tukey’s multiple comparisons test. Values of *p* < 0.05 were considered to be statistically significant.

## 3. Results

### 3.1. The MDR Group Sera Increased the Migration and Invasion of the HaCaT Cells by Activating SIRT1

**Figure 1:**
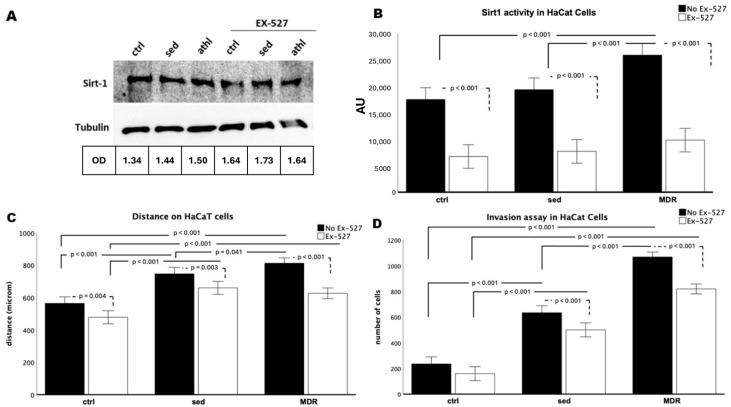
(**A**) Western blot analysis for SIRT1 obtained from protein extracts of HaCaT cells treated for 24 h with 10% *v*/*v* sera from sedentary people (sed) or athletes (MDR) with and without 15 µM of EX-527. Protein normalization was performed on the tubulin levels. (**B**) Evaluation of SIRT1 activity in HaCaT cells treated for 24 h with 10% *v*/*v* sera from sed or MDR groups with and without 15 µM of EX-527. (**C**) Results of the scratch wound healing assay on HaCaT cells treated or not treated with 10% *v*/*v* sed or MDR group sera and 15 µM of EX-527. (**D**) Results of the invasion assay. The bold line shows the comparison between the groups, while the dashed line shows the comparison in the presence or absence of EX-527.

The beneficial effect of exercise and the involvement of SIRT1 in skin wound healing are two phenomena that have already been characterized when considered separately. In this study, we evaluated how sera isolated from athletes practicing aerobic exercise (middle-distance running) could contribute to the activation of key cells involved in the main phases of tissue regeneration. First, human keratinocytes were conditioned, as previously reported, with sera isolated from athletes (the MDR group) or sedentary people (the sed group) [22] in the presence or absence of the selective SIRT1 inhibitor EX-527 [8,23]. While there were no changes in the expression levels of SIRT1 (Figure 1A), its activity was strongly increased in the HaCaT cells conditioned with the MDR group sera compared to that in both the control (ctrl) and sed-group-sera-conditioned cells (both *p* < 0.001). No difference was found between the ctrl and sed group sera cells in their SIRT1 activity levels (Figure 1B). As expected, in the presence of EX-527 with the sed or MDR group sera, the SIRT1 activity decreased compared to that in the ctrl, sed or MDR group sera in the absence of EX-527 (all *p* < 0.001, Figure 1B).

Then, as crucial events underlying re-epithelialization, we studied the migration and invasion capabilities of the HaCaT cells. As shown in Figure 1C, these keratinocytes acquired a high migration speed when treated with sed or MDR sera compared to that in the ctrl group (both *p* < 0.001). Interestingly, this event was more pronounced in MDR than in sed (*p* = 0.041). As shown in Figure 1C, a slowdown occurred in the ctrl plus EX-527 compared to the ctrl (*p* = 0.04), in sed plus EX-527 compared to sed (*p* ≤ 0.03) and in MDR plus EX-527 compared to MDR (*p* < 0.001). The invasion assay, measured through cell counting, revealed the acquisition of a higher cell speed in the presence of the sed and MDR group sera than that in the ctrl group (both, *p* < 0.001), with a more pronounced effect produced by MDR demonstrated by the difference between MDR and sed (*p* = 0.041). As shown in Figure 1D, the cell number decreased in the ctrl plus EX-527 compared to that in the ctrl without reaching statistical significance, in the sed group sera plus EX-527 compared to that in the sed sera alone (*p* < 0.001) and in the MDR group sera plus EX-527 compared to that in the MDR sera alone (*p* < 0.001). Notably, the cell number in the MDR plus EX-527 group remained higher compared to that in the sed (*p* < 0.001) and sed plus EX-527 groups, although without reaching statistical significance. Representative images of the wound healing and invasion assays are given in Figure S1.

### 3.2. The Levels of Motility and Differentiation Markers in the Human Keratinocytes Were Reversed in the Presence of the Selective SIRT1 Inhibitor

**Figure 2:**
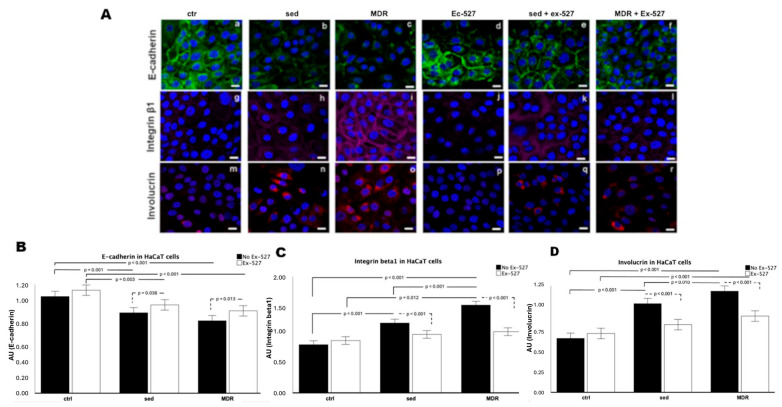
(**A**) Confocal analysis of HaCaT cells for E-cadherin (panels a–f), integrin β1 (panels g–l) and involucrin (panels m–r) after treatment for 24 h with 10% *v*/*v* sed group sera and MDR group sera with or without 15 µM of EX-527. Magnification of 63 × 1.4 NA. Bar = 20 μm. (**B**–**D**) Histograms showing fluorescence intensity analysis.

The activation state of the keratinocytes in the presence of human sera with and without the SIRT1 inhibitor EX-527 was assessed through a confocal analysis. First, we investigated the expression of E-cadherin to demonstrate the disengagement of cell–cell and cell–substrate junctions as one of the precursor events of migration [24]. The different levels of this protein confirmed the acquired ability of the cells to migrate more rapidly when treated with the MDR group sera, much more so than those treated with the sed group sera, with an inverse trend in the case of SIRT1 inhibition (Figure 2A, panels a–f). In detail, the E-cadherin expression appeared strongest in the unconditioned cells (Figure 2A, panel a) and in the presence of EX-527 (Figure 2A, panel d). It decreased more in MDR than in sed (Figure 2A, panels b and c). This reduction became less significant when EX-527 was added (Figure 2A, panels e and f).

We next evaluated the expression of integrin β1, as its action is crucial for cell migration during wound repair [25]. We found that the expression of this protein increased and was structured on the plasma membrane of the HaCaT cells in response to the MDR group sera, much more than in the cells conditioned with the sed group sera, compared to these properties in the unconditioned cells (Figure 2A, panels i, h and g, respectively). On the other hand, this effect on integrin β1 was reverted in the presence of EX-527 alone (Figure 2A, panels j, k, l). Finally, the detection of involucrin, as a precursor protein of the cornified envelope and a marker of terminal differentiation of the epidermal keratinocytes [26], demonstrated that in the presence of the MDR group sera, more than the sed group sera, the HaCaT cells appeared to be at a differentiated stage (Figure 2A, panels m, n and o).

In contrast, in the presence of EX-527, the involucrin expression strongly decreased (Figure 2A, panels p, q and r). These results were confirmed through a fluorescence intensity analysis (Figure 2B–D). In detail, the E-cadherin expression decreased in sed (*p* = 0.001) and MDR (*p* < 0.001) compared to that in the ctrl. The HaCaT cells conditioned with sed group sera plus EX-527 showed lower levels of E-cadherin than those in the ctrl plus EX-527 (*p* = 0.038). The cells conditioned with MDR plus EX-527 showed lower levels of E-cadherin than those in the ctrl plus EX-527. The presence of EX-527 induced an increase in E-cadherin expression compared to that in sed (*p* = 0.038) and MDR (*p* =0.013), whereas no effect on the E-cadherin expression was shown in the ctrl. The E-cadherin expression in the ctrl plus EX-527 was higher compared to that in sed plus EX-527 (*p* = 0.003) and MDR plus EX-527 (*p* < 0.001).

The integrin β1 expression increased in sed and MDR compared to the ctrl (both *p* < 0.001) and in MDR compared to that in sed (*p* < 0.001). The HaCaT cells conditioned with the sed group sera plus EX-527 showed lower levels of integrin β1 compared to those with the sed group sera alone (p < 0.001), with the same pattern found for with MDR group sera plus EX-527 compared to MDR group sera alone (*p* < 0.001). The cells conditioned with MDR plus EX-527 showed higher levels of integrin β1 than those in the ctrl plus EX-527 (*p* = 0.012).

The involucrin expression increased in sed and MDR compared to that in the ctrl (both *p* < 0.001) and in MDR compared to that in sed (*p* < 0.001). The HaCaT cells conditioned with sed group sera plus EX-527 showed lower levels of involucrin than those in the sed group sera alone (*p* < 0.001). The cells conditioned with MDR plus EX-527 showed lower levels of involucrin than those in the MDR group sera alone (*p* < 0.001). No difference was found between the ctrl plus EX-527 and the ctrl (Figure 2D).

### 3.3. Human Fibroblasts Acquired a Higher Rate of Migration and Invasion Speed When Treated with the MDR Group Sera, Depending on the Activity of SIRT1

**Figure 3:**
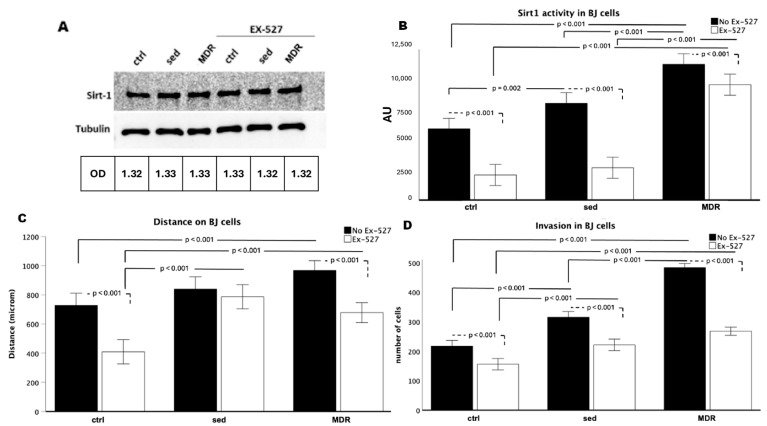
(**A**) Western blot analysis for SIRT1 obtained from BJ cells conditioned for 24 h with 10% *v*/*v* sera from sedentary people (sed) and athletes (MDR) with and without 15 µM of EX-527. Protein normalization was performed on tubulin levels. (**B**) Evaluation of SIRT1 activity in BJ cells conditioned for 24 h with 10% *v*/*v* sera from sed and MDR groups with and without 15 µM of EX-527. (**C**) Results of the scratch wound healing assay on BJ cells treated or not treated with the same experimental points. (**D**) Results of the invasion assay on BJ cells treated or not treated with the same experimental points.

We found that, as in the keratinocytes (the HaCaT cells), the expression of SIRT1 in the fibroblasts (the BJ cells) did not change (Figure 3A), whereas its activity was strongly affected by sera conditioning. Indeed, as shown in Figure 3B, the SIRT1 activity strongly increased in the cells conditioned with the MDR (*p* < 0.001) and sed (*p* = 0.002) group sera compared to that in the ctrl cells and in MDR compared to that in sed (*p* < 0.01). EX-527 induced a decrease in the SIRT1 activity in the ctrl, sed and MDR groups compared to that in the cells without the inhibitor (all *p* < 0.001). The cells treated with MDR plus EX-527 showed higher SIRT1 activity than that in the ctrl plus EX-527 and sed sera plus EX-527 groups (both *p* < 0.001).

Next, migration (Figure 3C) and invasion (Figure 3D) assays showed that the MDR group sera were able to induce fibroblast motility (MDR vs. ctrl = *p* < 0.001 for both migration and invasion). No difference was found in the migration between the sed and ctrl groups, whereas the invasion increased in sed compared to that in the ctrl (*p* < 0.001). Ex-527 induced a decrease in migration in the ctrl and MDR groups (both *p* <0.001) but not in the sed group and induced a decrease in invasion in the ctrl, sed and MDR groups (all *p* < 0.001). The migration was greater in MDR plus EX-527 than in ctrl plus EX-527 (*p* < 0.001) and in sed plus EX-527 than in the ctrl plus EX-527 (*p* < 0.001). No difference was shown between MDR plus EX-527 and sed plus EX-527. The invasion in MDR plus EX-527 was greater than that in the ctrl plus EX-527 (*p* < 0.001). Representative images of the wound healing and invasion assays are shown in Figure S2.

### 3.4. BJ Cell Activation Is Promoted by the MDR Group Sera and Inhibited by EX-527

**Figure 4:**
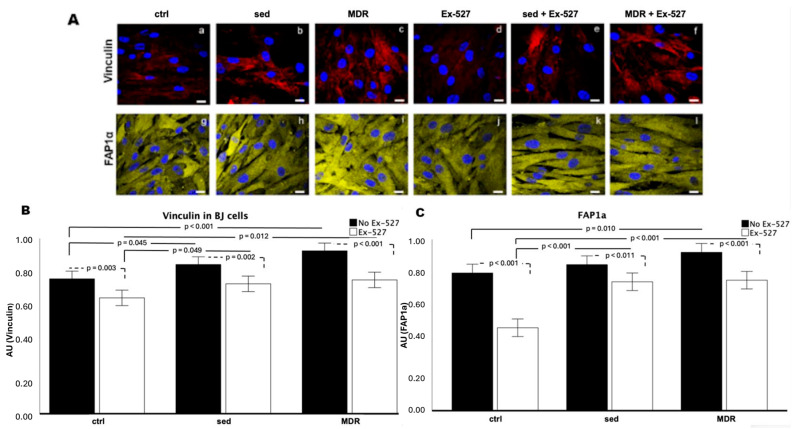
(**A**) Immunofluorescence assay on human fibroblasts for vinculin (panels a–f) and FAP1α (panels g–l) after treatments for 24 h with 10% *v*/*v* sera from sedentary people (sed) and athletes (MDR) in the presence or absence of 15 µM of EX-527. Magnification of 63 × 1.4 NA. Bar = 20 μm. (**B**) Analysis of the fluorescence intensity in vinculin histograms. (**C**) Analysis of the fluorescence intensity in FAP1α histograms.

The immunofluorescence assay on the fibroblasts (BJ cells) highlighted an increase in vinculin and FAP1α in the presence of the sed group sera and furthermore the MDR group sera compared to that in the ctrl group (Figure 4A, panels a–c and g–i). These two proteins were evaluated since the former is known to mediate the traction force generation exerted during motility [27], while the latter typically marks activated fibroblasts whose movement is particularly promoted in the stroma [28]. The assay revealed weak vinculin expression in both the absence and presence of EX-527 (Figure 4A, panels a and d).

The fluorescence intensity analysis revealed that the vinculin protein expression was higher in sed than that in the ctrl (*p* = 0.045) and in MDR compared to that in the ctrl (*p* < 0.001). In the presence of EX-527, the vinculin expression decreased in the ctrl, sed and MDR groups compared to that in their counterparts without the inhibitor (*p* = 0.003, *p* = 0.002 and *p* < 0.001, respectively). MDR plus EX-527 showed increased vinculin expression compared to that in the ctrl plus EX-527 (*p* = 0.012) (Figure 4B).

The FAP1α expression levels increased in the presence of the MDR group sera compared to those in the ctrl (*p* = 0.010) (Figure 4C). A reduction in the FAP1α expression levels occurred in the ctrl plus EX-527 compared to those in the ctrl (*p* < 0.001), in the sed group sera plus EX-527 compared to the sed group sera alone (*p* < 0.011) and in the MDR group sera plus EX-527 compared to the MDR group sera alone (*p* < 0.001) (Figure 4C). For both protein markers, no statistically significant differences were found between MDR plus EX-527 and sed plus EX-527.

### 3.5. Motility of Human Endothelial Cells Is Enhanced in the Presence of MDR Sera if SIRT1 Is Not Inhibited

**Figure 5:**
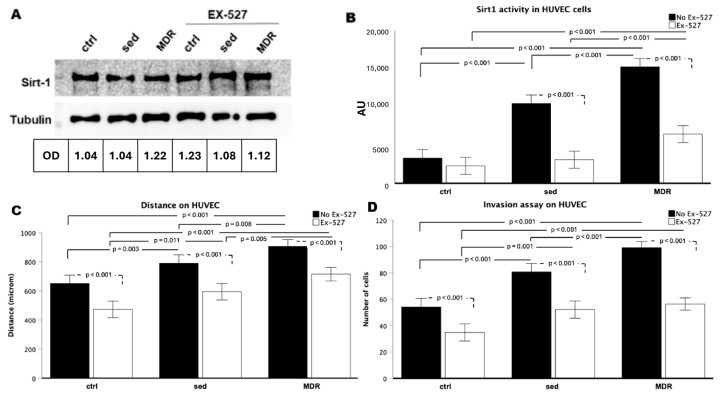
(**A**) Western blot analysis on HUVEC cells for SIRT1 protein after conditioning for 24 h with 10% *v*/*v* sera from sedentary people (sed) and athletes (MDR) in the presence or absence of 15 µM of EX-527. Protein normalization was performed on tubulin levels. (**B**) Evaluation of SIRT1 activity in HUVEC cells after conditioning for 24 h with 10% *v*/*v* sed and MDR group sera in the presence or absence of 15 µM of EX-527. (**C**) Results of scratch wound healing assay on HUVEC cells conditioned or not conditioned with human sera and treated or not treated with EX-527. (**D**) Invasion assay results for the same experimental points.

The HUVEC endothelial cells conditioned with human sera and treated with EX-527 showed similar results to those found in the keratinocytes and fibroblasts. In detail, while the SIRT1 expression never changed (Figure 5A), the SIRT1 activity appeared to be strongly influenced. In fact, it increased in sed and MDR compared to that in the ctrl (both, *p* < 0.001). Notably, the SIRT1 activity was higher in MDR than that in sed (*p* < 0.001). It decreased in the sed group sera plus EX-527 compared to that in sed group sera alone (*p* < 0.001) and in the MDR group sera plus EX-527 compared to that in MDR group sera alone (*p* < 0.001), whereas no difference was found between the ctrl plus EX-527 and the ctrl (Figure 5B).

We next found that the migration increased in sed (*p* = 0.003) and MDR (*p* < 0.001) compared to that in the ctrl. The cells conditioned with the MDR group sera showed higher levels of migration than that in the cells conditioned with the sed group sera (*p* = 0.008) (Figure 5C). As shown in Figure 5D, invasion was influenced by the sera conditioning as assessed by cell number, which increased in sed compared to that in the ctrl (*p* < 0.001) and in MDR compared to that in the ctrl (*p* < 0.001). Also, in this case, the increase was more pronounced in MDR than that in sed (MDR vs. sed, *p* < 0.001).

As shown in Figure 5C,D, in the presence of EX-527, cellular migration and invasion decreased in the ctrl, sed and MDR groups compared to these properties in their counterparts without the inhibitor (all *p* < 0.001). Representative images of the wound healing and invasion assays are given in Figure S3.

### 3.6. In Vitro Angiogenesis and CD31 Expression Are Amplified by MDR Group Sera but Not in the Presence of EX-527

**Figure 6:**
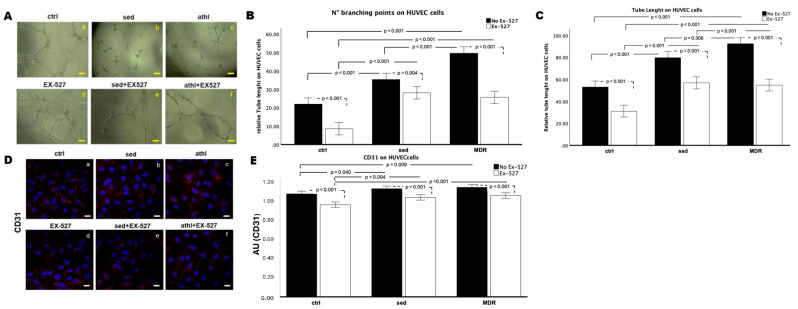
(**A**) Representative bright field images of in vitro angiogenesis (panels a–f) and (**B**,**C**) analysis of number of branches and tube length calculated using ImageJ (Angiogenesis Analyzer tool) software 2.14.0 version on HUVEC cells conditioned with 10% *v*/*v* sera from sed and MDR groups with and without 15 µM of EX-527. Bar = 100 µm. (**D**) A confocal analysis on HUVEC cells for CD31 after the same treatments (panels a–f). Magnification of 63 × 1.4 NA. Bar = 20 μm. (**E**) Histograms of the fluorescence intensity.

Once the motility of the endothelial cells had been tested, they were evaluated in terms of their ability to form capillary-like structures in vitro. Representative images of angiogenesis are shown in Figure 6A. A quantitative analysis was performed considering the number of branching points (Figure 6B) and the relative tube length (Figure 6C). The number of branching points tended to increase in sed and significantly increased in MDR compared to that in the ctrl (*p* < 0.001). The number of branching points decreased in the sed group sera plus EX-527 compared to that in the sed group sera alone, without reaching statistical significance, and decreased in the MDR group sera plus EX-527 compared to that in the MDR group sera alone (*p* < 0.001) and in the ctrl plus Ex-527 group compared to that in the ctrl group (*p* = 0.023). These values were higher in MDR plus EX-527 and sed plus EX-527 than the ctrl plus EX-527 (*p* = 0.031 and *p* = 0.015, respectively). No difference was found between MDR plus EX-527 and sed plus EX-527 (Figure 6B).

The relative tube length increased in sed and MDR compared to that in the ctrl (both *p* < 0.001) and in MDR compared to that in sed (*p* = 0.006) and decreased in the presence of EX-527 in the ctrl, sed and MDR groups compared to that in their counterparts without the inhibitor (all *p* < 0.001) (Figure 6C).

Finally, we assessed the CD31 expression, as it is one of the most characterized markers of activated endothelial cells [29]. The confocal analysis demonstrated that its levels were increased in sed compared to those in the ctrl (*p* = 0.040) and in MDR compared to those in the ctrl (*p* = 0.009).

The treatment with EX-527 reversed this condition. In fact, the CD31 expression decreased in the ctrl, sed and MDR plus EX-527 groups compared to that in the ctrl, sed and MDR groups without the inhibitor (all *p* < 0.001). No statistically significant difference between sed plus EX-527 and MDR plus EX-527 was found (Figure 6D,E).

## 4. Discussion

In previous studies, we demonstrated that serum isolated from triathletes practicing aerobic ET, and serum from patients with heart failure who underwent an aerobic-exercise-based cardiac rehabilitation program, was able to protect endothelial cells from oxidative stress and senescence and induce cell proliferation in a SIRT1-dependent manner [9,22]. In the context of cardiac rehabilitation based on regular aerobic ET, a significant correlation was observed between SIRT1 activity and serum hydroxybutyrate levels [30], which is recognized as a nanocomposite scaffold component for various wound healing applications [31,32].

In this study, we used the same ex vivo/in vitro approach, conditioning three cell lines (keratinocytes, fibroblasts and endothelial cells) mainly involved in the wound healing process with serum isolated from athletes who regularly participated in aerobic ET, i.e., MDR.

We recorded the changes in migration and invasion, as well as the expression of the protein markers analyzed, in all of the cell lines and the changes in angiogenesis in the endothelial cells after conditioning with the sed and MDR group sera. This suggested the ability of human serum to interfere with cell performance in general.

However, conditioning with the MDR group sera resulted in a greater increase in SIRT1 activity than that with the sed group sera. This is probably due to the fact that regular aerobic ET is associated with increased levels of NAD+, which is the enzymatic cofactor of SIRT1 [33,34,35]. At the same time, invasion, particularly in the keratinocytes and fibroblasts; migration in the keratinocytes and endothelial cells; and angiogenesis were more intensively promoted in the presence of the MDR group sera, and the selective inhibition of SIRT1 activity by EX-527 attenuated this effect. Overall, these results suggest that ET-induced SIRT1 plays a role in cellular events related to wound healing.

Our results have potential clinical implications, as SIRT1 is recognized as a promoter of cell survival, angiogenesis and cell migration and the stress response in conditions where wound healing is impaired, such as diabetes [14,36]. Indeed, pharmacological activation of SIRT1 has been shown to promote endothelial cell proliferation and angiogenesis and the epithelial–mesenchymal transition in keratinocytes and fibroblasts [14,36].

In the presence of the MDR group sera, compared to those with the sed group sera, in addition to an increase in SIRT1 activity, we observed higher levels of known differentiation markers such as involucrin and integrin β1 in the keratinocytes, FAP1α in the fibroblasts and CD31 in the endothelial cells (although to a lesser extent). These results confirm the involvement of SIRT1 in the differentiation process, as assessed in vivo through models of SIRT1 knockout mice that die during fetal development or immediately after birth due to severe developmental defects [37]. It has also been reported that SIRT1 promotes astroglial differentiation to facilitate astrogliosis and healing, which results in scarring at the damaged site, commonly observed in brain and spinal cord injuries [38]. Further data on the activity of SIRT1 in promoting chondrogenic development and the differentiation of skeletal myoblasts have been reported that frame this protein as an important promoter of cell differentiation, especially in response to certain stress conditions [39,40]. Moreover, a link between ET, SIRT1, tissue regeneration and repair has been found in the context of neurogenesis, myogenesis and osteogenesis [3]. For the first time, we have shown that ET-induced SIRT1 is involved in the molecular mechanisms underlying skin wound repair.

This study has several limitations. As we did not measure the NAD+ levels, we cannot be certain that the increase in SIRT1 activity was due to the increased availability of its cofactor. However, several in vivo and human studies agree that chronic exercise induces SIRT1 activity by increasing NAD+ [41,42,43]. In fact, as mentioned above, aerobic ET is considered one of the best strategies for increasing NAD+ levels [6,33], depending on the type and duration of ET, and can occur in tissues such as the skeletal muscle and in the blood through augmented hepatic synthesis of NAD+ precursors [6,35]. Although the enrolled athletes did not follow a particular diet or take herbal supplements during ET, it cannot be excluded that other factors may have induced a direct increase in SIRT1 or its cofactor NAD+. Furthermore, our results are based on ex vivo/in vitro experiments and need to be confirmed in vivo and in a human model.

## 5. Conclusions

This study demonstrates the significant impact of aerobic ET in promoting skin wound healing, highlighting the central role of SIRT1 in promoting the motility and differentiation of the keratinocytes, fibroblasts and endothelial cells.

This analysis of exercise-induced cellular activation encourages future research efforts to investigate other possible ET-related protective effects in the context of stress injuries that compromise the proper wound healing by identifying potentially involved circulating factors.

## Figures and Tables

**Table 1:** Characteristics of the study population.

	MDR (N = 23)	SED (N = 12)	*p*-Value
Age, years (mean ± SD)	55.35 ± 7.21	49.83 ± 9.75	0.07
Height (m)	1.72	1.67	0.12
Weight (kg)	69.17	77.75	0.033
BMI (mean ± SD)	23.47 ± 2.37	27.28 ± 3.77	0.00083
Weekly frequency of training, times/week (mean ± SD)	3.32 ± 0.89	-	-
Kilometers travelled per week (mean ± SD)	38.21 ± 16	-	-

Abbreviations: MDR, middle-distance running; SED, sedentary volunteers; SD, standard deviation; BMI, Body Mass Index.

## Data Availability

The datasets used and/or analyzed during the current study are available from the corresponding author on reasonable request.

## Supplementary Materials

The following supporting information can be downloaded at: https://www.mdpi.com/article/10.3390/biomedicines13051041/s1. Figure S1: (A) Representative images of HaCaT cells in the wound healing assay at T0h and T24h. (B) Representative images of the same cells in the invasion assay. Bar = 100 µm. Figure S2: (A) Representative images of BJ cells in the wound healing assay at T0h and T24h. (B) Representative images of the same cells in the invasion assay. Bar = 100 µm. Figure S3: (A) Representative images of HUVEC cells in the wound healing assay at T0h and T24h. (B) Representative images of the same cells in the invasion assay. Bar = 100 µm.

## Abbreviations

ET	exercise training
NAD+	nicotinamide adenine dinucleotide
SIRT1	sirtuin 1
MDR	middle-distance running
sed	sedentary volunteers
HUVEC	human umbilical vein endothelial cells
ctrl	control
BIO-RAD	Bio-Rad Dye Reagent Concentrate
BSA	bovine serum albumin
SD	standard deviation
