# Supplementary material for: SIRT1 Mediates the Effects of Sera from Athletes Who Engage in Aerobic Exercise Training in Activating Cells for Wound Healing
Source: Biomedicines. 2025 Apr 25;13(5):1041. doi: 10.3390/biomedicines13051041 (PMC12108720; doi:10.3390/biomedicines13051041)
Supplement: Supplementary file 1 [file biomedicines-13-01041-s001.zip › biomedicines-3590032-supplementary.pdf]

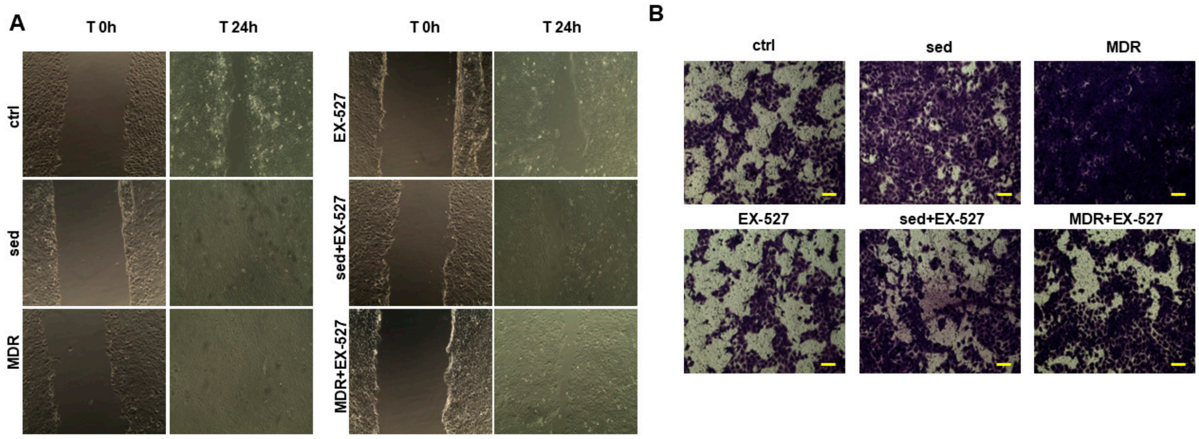

Figure S1. (A) Representative images of HaCaT cells in the wound healing assay at T0h and T24h. (B) Representative images of the same cells in the invasion assay. Bar = 100 µm.

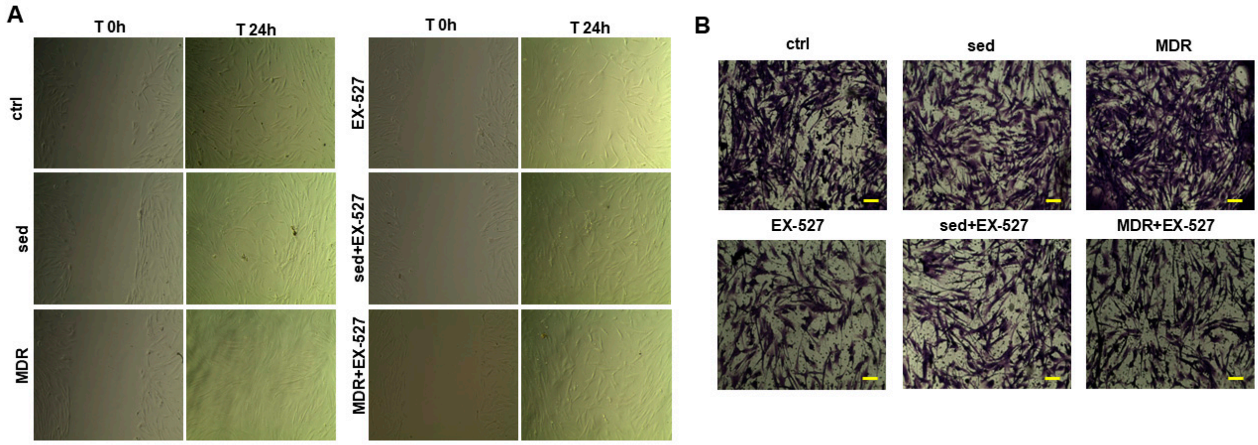

Figure S2. (A) Representative images of BJ cells in the wound healing assay at T0h and T24h. (B) Representative images of the same cells in the invasion assay. Bar = 100 µm.

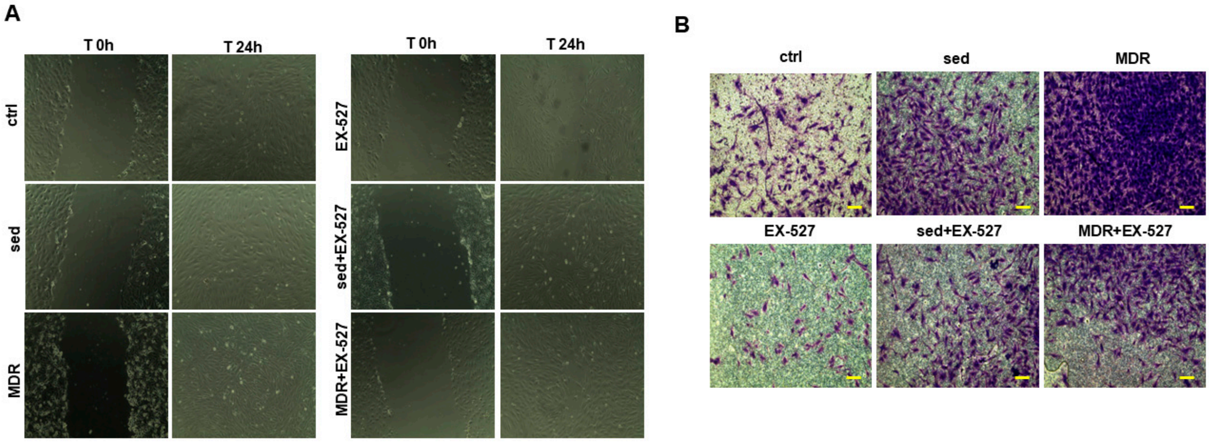

Figure S3. (A) Representative images of HUVEC cells in the wound healing assay at T0h and T24h. (B) Representative images of the same cells in the invasion assay. Bar = 100 μm.
